# Retrotransposition of R2 elements in somatic nuclei during the early development of *Drosophila*

**DOI:** 10.1186/1759-8753-2-11

**Published:** 2011-09-29

**Authors:** Michael T Eickbush, Thomas H Eickbush

**Affiliations:** 1 Department of Biology, University of Rochester, Rochester, NY, USA; 2 Fred Hutchinson Cancer Research Center, Seattle, WA, USA

## Abstract

**Background:**

R2 retrotransposable elements exclusively insert in the 28S rRNA genes of their host. Their RNA transcripts are produced by self-processing from a 28S R2 cotranscript. Because full-length R2 transcripts are found in most tissues of R2-active animals, we tested whether new R2 insertions occurred in somatic tissues even though such events would be an evolutionary dead end.

**Findings:**

PCR assays were used to identify somatic R2 insertions in isolated adult tissues and larval imaginal discs of *Drosophila simulans*. R2 somatic mosaics were detected encompassing cells from individual tissues as well as tissues from multiple body segments. The somatic insertions had 5' junction sequences characteristic of germline insertions suggesting they represented authentic retrotransposition events.

**Conclusions:**

Body segments are specified early in *Drosophila *development, thus the detection of the same somatic insertion in cells from multiple tissues suggested that the R2 retrotransposition events had occurred before the blastoderm stage of *Drosophila *development. R2 activity at this stage, when embryonic nuclei are rapidly dividing in a common cytoplasm, suggests that some retrotransposition events appearing as germline events may correspond to germline mosaicism.

## Findings

Mobile element insertions during the development of somatic tissues provide no benefit to the element, as these insertions are not transferred to subsequent generations. Thus in animals, where the separation of somatic and germline tissues is established early, the ability of a mobile element to generate new insertions in somatic tissues would most likely be selected against. Consistent with this prediction, early studies in *Drosophila melanogaster *showed that P element transpositions were dependent upon a germline-specific RNA splicing component [1], and I elements were only transcribed in ovaries [2]. However, counter to this model, mobile elements in other animals have been shown to generate new insertions in somatic tissues (for example, Tc1 elements in *Caenorhabditis elegans *[3], L1 elements in mammals [4,5]).

Several explanations can be put forward for the somatic activity of mobile elements. First, somatic events are inconsequential to the host and thus there is little selective pressure for a mobile element to evolve specificity to the germline. Second, somatic events are harmful, however it is risky for a mobile element to become dependent on a germline-specific mechanism, as it provides another opportunity for the host to control the element. Third, on occasions somatic events provide a benefit to the host. This last fascinating possibility has been suggested to explain the ability of L1 to retrotranspose in nerve tissues [6].

**Figure 1:**
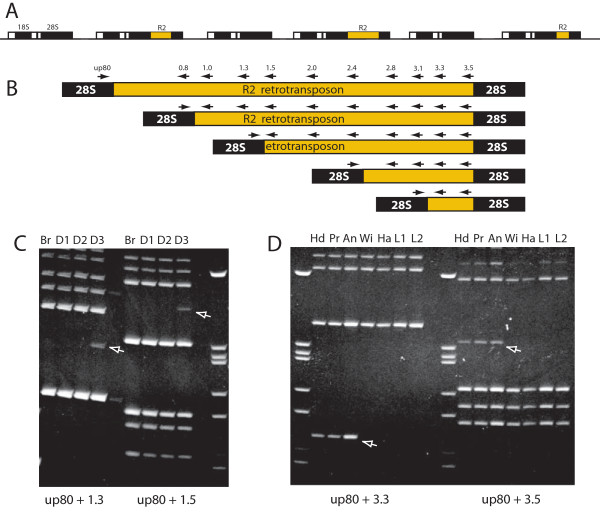
**Diagram of R2 insertions within the rRNA genes of *Drosophila *and the PCR assay used to monitor somatic mosaicism**. **(A) **Diagram of the tandemly repeated rRNA genes of *Drosophila simulans *and the location of R2 insertions. Black boxes, 18S, 5.8S and 28s rRNA genes (5.8S gene between the 18S and 28S genes is not labeled); white boxes, transcribed spacer regions. **(B) **About half of the R2 insertions have deletions of their 5' end that can extend to nearly the entire length of the element. All R2 copies have the same 3' junction with the 28S gene. Arrows above the R2/28S diagrams indicate the positions of the oligonucleotide primers used to assay for the 5' truncations. The DNA extraction method, the primers used and the PCR protocols used were identical to those in previous reports [11-13]. **(C) **Examples of the ethidium stained PCR products derived from larval tissues. The larval tissues were dissected in *Drosophila *Ringers. **(D) **Examples of the ethidium stained PCR products derived from adult tissues. Adult tissues were placed directly in the DNA extraction solution. PCR bands interpreted as somatic insertions are indicated with arrows. To be scored as a somatic mosaic the amplified band had to be detected with two sets of primer combinations (shown below the figures). The following abbreviations for body segments were used: An = antenna; Br, brain from a larvae; D1-D3, individual pairs of imaginal disc (the specific disc pair used was not known); Ha = haltere; Hd = head; L1 and L2 = individuals legs from different body segments; Pr = proboscis; Wi = wing.

R2 non-LTR retrotransposable elements specifically insert into the tandemly repeated rRNA genes of many animal genera, Figure 1A[7,8]. Each R2 insertion blocks the production of functional 28S rRNA from the inserted gene. Because animals contain many more rRNA genes than are needed for transcription [9,10], in most individuals inserted rRNA units are simply not transcribed. However, studies in *Drosophila simulans *indicate that in individuals where R2-inserted units are distributed throughout the rDNA locus, inserted rDNA units are transcribed [11-13]. The R2 transcripts are processed from the cotranscript [14], and new germline retrotransposition events can be observed in the progeny. Because rRNA transcription is essential in all tissues, and full-length R2 transcripts are readily detected in most larval and adult tissues of active lines ([12] and D. Eickbush and T. Eickbush, unpublished results), we tested whether R2 retrotranspositions also occur in somatic tissues. The *D. simulans *stock selected for study, sim89, had high levels of R2 transcripts in many tissues and new insertions could be detected in progeny that had originated in either the male or female parent [12,15]. For each animal the screens for R2 somatic mosaics were conducted with either seven adult tissues (antenna, proboscis, the rest of the head, wing, haltere and individual legs from different thoracic segments), or with four third instar larval tissues that are precursors to adult tissues (brain, and three pairs of imaginal discs). Larval-specific tissues were not used because they are composed of polytene cells, which under-replicate R2-inserted rDNA units [16]. New insertions were assayed using the same 5' junction PCR assays previously used to detect germline events [11,15,17]. These assays utilize the property that while all R2 insertions occur at the identical location in the 28S genes when monitored from the 3' end, over half of the R2 retrotransposition events result in a deletion of element sequences starting at its 5' end and extending to locations throughout its 3.6 kb length. These 5' truncated copies can also contain short deletions or duplications of upstream 28S gene sequences. As a result somatic R2 insertions containing 5' truncations will generate PCR bands of lengths that seldom match the lengths of the PCR bands derived from the germline inherited 5' truncated elements. The PCR assays were conducted using a single primer located 80 bp upstream of the R2 insertion site in combination with a series of primers to sequences spaced throughout the length of the R2 element (Figure 1B) [12].

Somatic mosaics were defined as the presence of unique PCR bands in only a subset of the tissues tested from a single animal. To be scored as a somatic insertion, each new PCR band also had to be reproducibly detected using two different PCR primer combinations. Examples of an R2 insertion in one tissue of the four tested from a third instar larva, and of another insertion detected in three of seven adult tissues are shown in Figure 1C, D. The PCR bands representing potential somatic events were less intense than the bands derived from the R2 elements inherited from the mother or father, as expected if not all cells of a tissue type contained the insertion. Generally new bands could be reproducibly observed if they corresponded to at least one-tenth the intensity of those bands derived from inherited R2 copies. In total, tissues from 29 individuals (14 females, 15 males) were scored. A total of 15 potential somatic insertions were detected in 7 animals (2 females with 4 total events, and 5 males with 11 total events). The detection of greater numbers of new insertions in males compared to females was likely due to the greater sensitivity of the PCR assay in males. The rRNA genes in *D. simulans *are located on the × chromosome [18]: thus males contain a single rDNA locus, compared to two copies of the rDNA locus in females. The somatic events were detected in essentially all tissues examined, although the numbers of events were not sufficient to make conclusions about relative frequencies.

**Figure 2:**
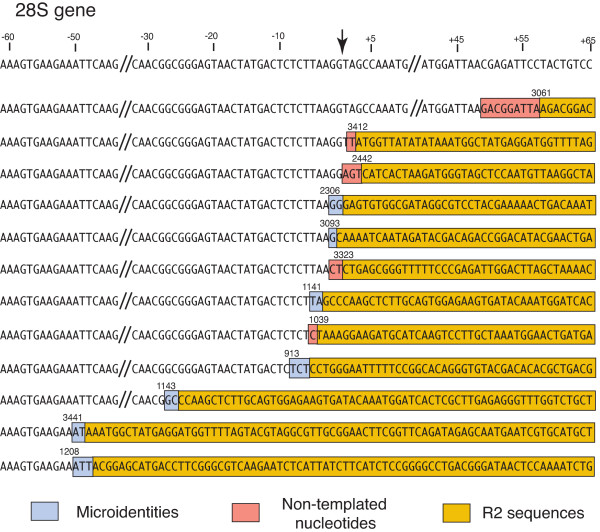
**Diagram of the 5' junction sequences of the somatic R2 insertions with the 28S gene**. Putative somatic insertions such as those shown in Figure 1C, D were excised from a gel, re-amplified with the same PCR primers, purified on a second gel and subjected to double-stranded DNA sequencing. The 28S gene sequence is shown at the top of the figure. Short regions upstream and downstream of the R2 insertion site (arrow) are not shown because no R2 junctions occurred in these areas. For each junction those sequences corresponding to R2 sequences have been highlighted with tan shaded boxes. Those nucleotides that could correspond to either the 28S sequence or R2 (described as microidentities in the text) have been indicated with a blue box. Those sequences that do not correspond to either the 28S gene or R2 (described as non-templated nucleotides in the text) are indicated with an orange box. The number at each junction corresponds to the first nucleotide of the R2 element, based on the consensus *Drosophila simulans *R2 sequence [18].

To confirm that the PCR bands detected in only a subset of tissues corresponded to new R2 insertions arising from retrotransposition mechanisms similar to that of germline events, PCR bands representing 12 events that were well separated from the germline bands were excised from the gel, reamplified and the product sequenced. As shown in Figure 2 the 5' ends of the somatic R2 insertions had the characteristics associated with germline R2 insertions [19]. First, the 5' junctions of the R2 sequences with the 28S gene occurred at a variety of positions near the R2 insertion site. As with germline insertions, most junctions were within a few base pairs of the insertion site, but a few were found at distances of approximately 25 and 50 nucleotides. Second, seven insertions had microidentities of from 1-3 nucleotides between the R2 element and the upstream 28S sequence (sequences highlighted in blue). These microidentities are suggested to arise by the R2 DNA polymerase (also known as reverse transcriptase) annealing the upstream target DNA of the 28S gene to the newly made cDNA strand to prime second strand DNA synthesis. Microidentities at the 5' junction of truncated copies is a common property of L1 and other non-LTR retrotransposons [20]. Third, in those cases with no microidentity, from 1-9 nucleotides were present at the junction that did not correspond to either the upstream 28S gene or the R2 element (sequences highlighted in orange). These bases are postulated to represent non-templated synthesis by the R2 reverse transcriptase on the second DNA strand cleavage site until a microidentity between these added nucleotides and the cDNA strand enables the polymerase to prime second strand DNA synthesis. In summary, the physical properties of the 5' junctions of the new PCR bands detected in somatic tissues suggest they represent authentic retrotransposition events.

Because the development of *Drosophila *has been intensively investigated, the timing of the retrotransposition events that generated the observed somatic mosaics can be estimated. By mid-embryogenesis (10-12 h), small clusters of cells (10-40 cells) are specified to become individual imaginal discs [21,22]. Each imaginal disc primordium divides during the 3 larval instars to form from 10,000 to 60,000 cells by late larval development [23]. Because the observed somatic events were present in a significant fraction of the cells present in a third instar larval disc or an adult tissue, the retrotransposition events probably occurred before or early in imaginal disc development. Those retrotransposition events detected in more than one disc or adult appendage probably occurred even earlier in development, before determination of body segments at the blastoderm stage (2-3 h). Of the 15 events we observed, 5 were detected in cells derived from more than 1 body segment. Because we surveyed only a fraction of all body segments in either the larvae or adult, it is likely that a larger fraction of the somatic R2 insertion events we observed occurred before the blastoderm stage. This developmental period corresponds to rapid nuclear division in a common cytoplasm. During this period there is little RNA synthesis but active protein synthesis using the RNA synthesized by the nurse cells and deposited in the oocyte during oogenesis [24]. Because rRNA synthesis also does not occur in these first hours of development [25], R2 retrotransposition events occurring during this time probably use RNA templates synthesized by the nurse cells during oogenesis.

It should be noted that the observed somatic retrotransposition events likely occurred at a time when embryonic nuclei had not yet entered the pole plasma of the egg to become the germline. Thus in addition to somatic mosiacism there is also likely to be germline mosiacism of R2 elements in *Drosophila*. As a result, re-evaluation of a previous study of retrotransposition in the germline of males and females appears warranted [15]. We have previously suggested that the rate of R2 insertion inherited through the male germline was one-third to one-quarter the rate of insertions through the female germline. Based on the findings in this report, it is possible that all of the insertions scored as inherited through the male germline (that is, during spermatogenesis), actually occurred during early embryogenesis.

Because preblastoderm development in male and female embryos are similar, we suggest the higher rate of R2 insertions observed through the female germline represents this germline mosiacism as well as authentic germline events during oogenesis. Two separate periods of R2 activity in females was also consistent with experiments to monitor a large fraction of the offspring from individual females. In the most comprehensive study, new insertions were assayed in 213 progeny of a single female [15]. Of the 32 different R2 insertions detected in these progeny, 27 were found in only 1 individual and 4 were detected in 2 individuals. These insertions appeared to have occurred late in the development of the germline (that is, during oogenesis). The final R2 insertion was detected in 13 progeny, and could correspond to an insertion during early development. Additional evidence for germline mosiacism was found in the analysis of progeny from another female in which 6 of 17 individuals contained the same new R2 insertion.

In conclusion, we suggest that R2 elements are active early in *Drosophila *development, and as in the case with L1 elements in mouse and humans [4,5], can lead to both somatic and germline mosiacism. To determine if R2 elements are also active in somatic tissues later in development will require assaying many smaller samples from individual tissues or more sensitive approaches to detect insertions in smaller percentages of cells. Finally, R2 should serve as a reminder in the study of other mobile elements that events early in development can give rise to insertion mosaics that could be misinterpreted as germline events in the subsequent generation.

## Competing interests

The authors declare that they have no competing interests.

## Authors' contributions

MTE helped design the experiments, conducted all the experiments, and help perfect the manuscript. THE helped design the experiments and wrote the first draft of the manuscript. Both authors read and approved the final manuscript.
